# Collaborative Care Models of Primary Care Clinics for People with Early-Stage Dementia: A Cross-Sectional Survey of Primary Care Physicians in Japan

**DOI:** 10.5334/ijic.7726

**Published:** 2024-06-03

**Authors:** Shuji Tsuda, Junichiro Toya, Kae Ito

**Affiliations:** 1 Tokyo Metropolitan Institute for Geriatrics and Gerontology, 35-2 Sakae-cho, Itabashi, Tokyo 173-0015, Japan; 2 Sakurashinmachi Urban Clinic, 3-21-1-2F Shinmachi, Setagaya, Tokyo 154-0014, Japan

**Keywords:** dementia, early-stage, primary care, case management, collaborative care

## Abstract

**Objectives:**

This study explored collaboration models between primary care physicians (PCPs) and care managers (CMs) and assessed each model’s potential in meeting the support needs of individuals with early-stage dementia.

**Methods:**

In 2022, a cross-sectional survey was conducted among the PCPs in Tokyo. The data regarding the participant and clinic characteristics and daily practices for individuals with early-stage dementia were collected. The clinical collaborative practice was classified using a latent class analysis; comparisons were made between the identified classes based on 14 items in seven domains of support.

**Results:**

Two collaborative and one stand-alone models were identified. The former varied in the professionals’ roles, with one led by PCPs and the other by CMs. We named them PCP-led, CM-led, and stand-alone models, accounting for 46.4%, 32.8%, and 20.6% of the clinics, respectively. The PCP-led clinics were significantly more likely to provide support than the stand-alone ones across five domains: cognitive function, care planning, carers’ support, information, and social health. The CM-led model clinics generally fell between those of the other two models.

**Conclusion:**

Different leadership styles exist in the PCP-CM collaborations in care delivery for people with early-stage dementia. This collaboration offers distinct advantages for clinics in addressing their needs.

## Introduction

The increasing capacity of medical systems to diagnose dementia at an early stage highlights the urgent need to establish a system to provide support immediately after diagnosis [1,2]. Support should prioritise maintaining and enhancing the well-being of individuals diagnosed with dementia as well as their carers [3]. To achieve this goal, it is essential to address the unique support needs arising from dementia that can affect various aspects of a person’s life, including physical, psychological, cognitive, and social health [4]. A collaborative approach involving different care providers is necessary to ensure that the support-provision system accommodates diverse and complex needs [2,5]. The arena for implementing the dementia care system has been shifting from specialised to primary care, where primary, social, and long-term care (LTC) providers in the community collectively work towards this goal [6].

As dementia progresses and cognitive and physical functions decline, the support needs of the affected individuals and their families change accordingly [7]. During the moderate and severe stages, where declining functions can lead to the behavioural and psychological symptoms of dementia (BPSD) and activity limitations in daily life, common support needs include care for BPSD, physical nursing care, practical support for domestic life, and caregiver support. The existing services meet these needs relatively well [4]. However, there are distinct support needs in the early stage regarding the psychological impact of receiving a dementia diagnosis, including the acceptance/denial of diagnosis and managing anxiety as well as addressing social issues like loss of social connections and activities [8]. The most frequently unmet needs reported by individuals with dementia are psychological distress, access to information, engagement in daytime activities, and companionship [4]. Individuals newly diagnosed with dementia often regret not receiving multiple sessions until they grasp the meaning of the diagnosis and cope with the condition [9]. As these examples suggest, early-stage support needs are likely to remain unmet.

A promising healthcare delivery model for dementia care is case management, a collaborative and proactive approach aimed at providing coordinated and comprehensive care to patients with complex healthcare needs [2,10]. This involves systematic assessment, care planning, and care coordination to integrate services around individual needs [10,11].

Various approaches to implementing dementia case management in primary care settings have been explored, including integrated models and primary care physician (PCP)-case management partnership models [12,13]. An example of integrated models is primary care-based memory clinics, which incorporate dementia-specialised teams embedded in primary care clinics, providing readily available specialist consultations and case management to PCPs working with patients with dementia in any stages. These models have been shown to be particularly beneficial for collaborative medical management between specialists and PCPs, such as giving timely and appropriate diagnoses and engaging in the pharmacological management of BPSD [14,15]. Though team members in these models have perceived the model to have enhanced the provision of interprofessional care to each patient [16], an evaluation of the outcomes of the model did not show consistent benefits to patients’ overall quality of life or carers’ caregiving burden [17].

PCP-case management partnership models leverage collaboration between PCPs and case managers (CMs) within or across organisations to serve as an interdisciplinary liaison to meet individuals’ complex needs dispersed across different categories, including medical, long-term, and social care, while preventing the fragmentation of care across providers [5,18,19]. Abundant evidence supports the positive impacts of this model, such as BPSD management, delaying nursing home placement, and reducing the caregiving burden [13,20,21]. However, many of these studies did not specify the stage of dementia or focused on the moderate-to-late stages [11]. When examining evidence specific to the case management for early-stage dementia, insufficient research articles are available; further, some studies have found no superiority in the model [22,23]. As an early diagnosis of this progressive condition becomes increasingly possible, it is crucial to examine whether case management based on the PCP-CM collaboration can effectively accommodate the needs of individuals at an early stage [11,24].

In 2006, the Japanese government enacted legislation mandating the construction of the Community-based Integrated Care System to provide appropriate living arrangements, social care, and daily life support services within communities [25]. Under this legislation, municipal governments are responsible for managing the system to integrate prevention and medical and LTC services to address the needs of residents, including those with dementia. In addition to establishing the Community-based Integrated Care System, the Japanese government has provided financial incentives for primary care clinics and LTC providers to collaboratively offer home-based care in response to the growing public desire to live at home until the end of life [26]. This has created a driving force for practising PCP-case management partnership model of dementia care through collaboration between the PCPs in clinics and the CMs located within LTC organisations in the community. Medical and LTC insurance systems, which regulate each organisation’s activities, specify reimbursed collaboration mechanisms, including the exchange of formatted information and care plans, as well as participation in regular care conferences. This collaboration has been principally applied to the provision of care for people in the moderate and late stages of dementia, who are typically home-bound [27]. PCPs oversee collaborative medical care with other healthcare providers, such as home-visit nurses, while CMs coordinate non-medical homecare services, including assistance with daily activities at home, day care services, and respite care services.

The scope of the Community-based Integrated Care System also incorporates preventive social care for people with fewer disabilities, such as group-based exercise programs and dementia cafés. Community-based Comprehensive Support Centres (CCSCs) play a pivotal role by conducting individual needs assessments and support coordination with the involvement of community nurses, social workers, and CMs [26]. The PCPs are expected to refer patients with early-stage dementia to CMs based in the CCSCs to coordinate preventive social care services, indicating that the system may provide a foundation for PCP-case management partnerships in the early stage of dementia care. However, PCPs’ contribution to this service is not determined or reimbursed by the insurance systems [28]. Moreover, given that PCPs and CMs are typically situated in separate locations without electronic patient data sharing systems, effective communication between them necessitates additional effort. Thus, in the case of individuals with early-stage dementia, who are a targeted population for preventive social care from the CCSCs, case management can be applied, however, it may not rely on adequate collaboration between the PCPs and CMs. A previous study found a 17-month gap in the care that individuals experience before reaching out for appropriate social support after being newly diagnosed with dementia, despite social support resources being deployed in the community [29]. One of the reasons for this suboptimal use of available resources could be poor PCP-CM collaboration.

Given the limited evidence supporting the value of case management based on the PCP-CM collaboration for early-stage dementia care and insufficient knowledge about its implementation in the community, we examined the Japanese primary care practices for early-stage dementia care in which PCP-CM collaboration is readily available. This study aimed to examine: 1) whether and how community-based collaboration has been practised for individuals with early-stage dementia, and 2) the potential of the PCP-CM collaboration in meeting the diverse support needs of such individuals.

## Methods

### Study design and ethical procedures

This cross-sectional study analysed the survey data obtained from the PCPs working in clinics located in Tokyo Prefecture. The study materials and procedures were approved by the Ethics Committee of Tokyo Metropolitan Institute for Geriatrics and Gerontology (Approval number: R22–029). The survey was accompanied by written information about the research, and all participants provided written informed consent prior to their participation.

### Setting and participants

We administered a postal survey to Certified Dementia Support Doctors working in primary care clinics in Tokyo Prefecture. These doctors received endorsement from the regional medical association to which they belonged and had completed a governmental training course on community-based dementia care. The training course, consisting of an initial two-day course and optional follow-up courses, was launched by the Ministry of Health, Labour, and Welfare in 2005 as part of its dementia policy [30]. The training course covers diagnosis and pharmacological management of dementia and BPSD as well as psychosocial care for people with dementia and interdisciplinary collaboration in the community. In addition to working as PCPs or specialists for people with dementia in their primary work settings, trained doctors are expected to support and educate the PCPs and other care professionals working in the community and establish interprofessional liaison systems within the CCSCs. These systems include an interdisciplinary outreach service, the Initial-phase Intensive Support Team for dementia, which offers comprehensive assessment and coordination of care around the phase of dementia diagnosis [31].

**Figure 1:**
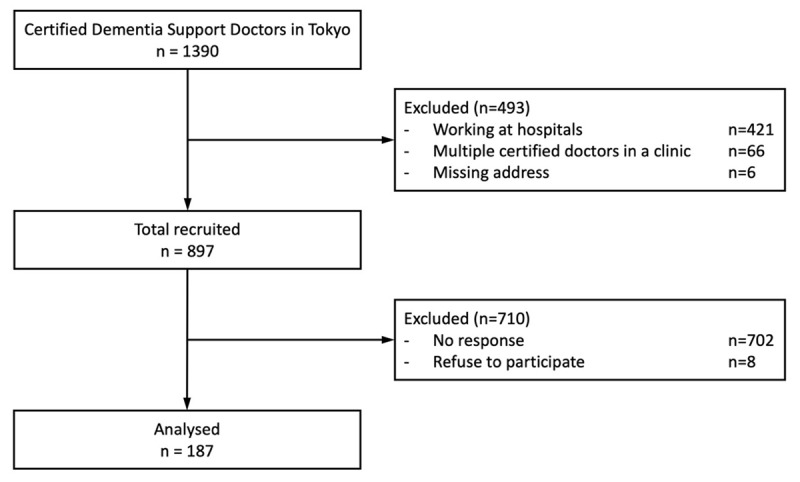
Flowchart of the participants.

Of the 1,390 Certified Dementia Support Doctors in Tokyo Prefecture in 2022, we excluded those who worked at hospitals and the Medical Centres for Dementia (hospitals assigned to provide specialised dementia care by the prefectural governments), resulting in the overall inclusion of 897 PCPs who worked at clinics at the time of the survey (Figure 1).

### Survey procedures

The survey was conducted between 1 October and 15 November 2022. The questionnaire was included in a packet containing a sponsorship letter from the certification course organiser and an invitation letter to one of the follow-up courses. We adopted the following measures to improve the participation rate: sending a postal mail reminder one week after completing the questionnaire and a second copy of the questionnaire three weeks later [32].

### Questionnaire and measures

We drafted the original survey questionnaire by referring to research articles and textbooks on primary and community-based integrated care for people with dementia [13,33,34]. To ensure the content validity of the questionnaire, we sought feedback from a panel of community-based dementia care experts including three PCPs and two psychiatrists. Each expert was given a single opportunity to comment on the clarity and relevance of the questions, particularly on the items listed for daily practices for individuals with early-stage dementia. We refined the question wording and revisited the list of items, incorporating their feedback.

We provided the following definition of early-stage dementia using the categories in the attending physician’s report for the LTC insurance that were familiar to the participants: early-stage dementia refers to patients who exhibit some symptoms of dementia but who are able to function independently in their daily domestic and social activities as well as patients who experience difficulties in performing daily tasks but who nevertheless are largely able to function independently with supervision.

#### Characteristics of the participants and their clinics

The questionnaire included questions on the participants’ characteristics, including age, sex, speciality, and membership in an outreach team (Initial-phase Intensive Support Team for dementia) in each community.

We also assessed the characteristics of their clinics, including the human resources (PCPs, nurses, social workers, CMs, rehabilitation therapists, and psychologists), number of patients with early-stage dementia, and average duration of consultations (with the PCPs, nurses, social workers, and CMs).

#### Variables for the classification of the practice models

To investigate whether and how the clinics applied community-based collaboration, we classified their practice models for early-stage dementia based on the individual- and organisation-level team configurations, roles, and communication regarding seeing patients and decision-making [34]. At the individual level, we collected data on the number of professionals involved in the team practice and the decision-making responsibilities of the PCPs and CMs for patients with early-stage dementia. At the organisational level, we investigated which organisations hosted care conferences and had the primary responsibility for making care decisions for each patient. In addition, we examined the inter-organisational communication between the clinics and other organisations (the Medical Centres for Dementia, CCSCs, and LTC offices) in the Community-based Integrated Care System.

#### Daily practices of support provision

We devised a comprehensive list of community-based support services available for individuals with early-stage dementia and their carers using the literature as a reference guide [33,34] and incorporating feedback from experts. The list comprised 49 items categorised into seven domains: care planning, informational support, cognitive function, psychological health, physical health, social health, and carer support. To enhance readability, this report presents the results of the first two items from each domain, with the remaining items displayed in the appendix table.

The questionnaire asked the participants about their daily practices regarding each item in the clinic, with three response options: offering the item in the clinic, referring the patient to another organisation that offered the item, or not covering it. To facilitate the analysis, we dichotomised the responses into two categories (offering/referring vs not covering), as the first two options indicated that the patients could receive the service.

### Analysis

We first reported the individual-level summary statistics of the participants’ characteristics, followed by an analysis of their clinic-level responses. The individual-level statistics included mean, standard deviation, frequency, and percentage. We utilised the R package entitled ‘Polytomous Variable Latent Class Analysis (poLCA)’ to perform a latent class analysis to classify the community-based collaborative practice models of the clinics [35]. The authors collectively examined the distribution of the modelling parameters to name each model. Accordingly, we drew on our knowledge of the clinics to ensure that the given names accurately reflected their practices. After assigning each clinic to a latent class based on the highest predicted probabilities derived from the observed responses, we compared the characteristics of the clinics and their daily support provision practices across the different classes. The categorical variables were analysed using a pair of chi-square tests to assess the overall differences and z-tests for pairwise comparisons. The continuous variables were assessed using the one-way analysis of variance and two-sample t-tests, and the ordinal variables were evaluated using the Kruskal–Wallis and Mann–Whitney U tests. To interpret the p-values, we set the level of significance at an alpha of 0.05 and applied the Bonferroni method to adjust for multiple comparisons. Patients with missing data were excluded from the analysis. All computations were performed using R ver. 4.2.2.

## Results

Of the 897 Certified Dementia Support Doctors who worked as PCPs, 195 responded to the survey, resulting in a response rate of 21.7%. After excluding those who refused to participate, 187 valid responses were included in the final analysis (Figure 1). A significant proportion of the PCPs were in their 50s (32.1%) and 60s (35.3%); furthermore, 79.1% were male. Regarding specialities, internal medicine, psychiatry, and family medicine accounted for 70.6%, 11.2%, and 4.3%, respectively (Table 1).

**Table 1:** Sample characteristics.


		n = 187	%

Age	30–39	5	2.7%

	40–49	24	12.8%

	50–59	60	32.1%

	60–69	66	35.3%

	70–99	30	16.1%

Sex	Men	148	79.1%

	Women	37	19.8%

Specialty	Internal medicine	132	70.6%

	Psychiatry	21	11.2%

	Family medicine	8	4.3%

	Orthopaedic surgery	6	3.2%

	Neurosurgery	6	3.2%

	Surgery	5	2.7%

	Other	9	4.8%

IIST	Yes	61	32.6%


IIST, Initial-phase Intensive Support Team.

We conducted a latent class analysis of the clinics’ collaborative practices and identified a three-class solution with the best goodness of fit. Based on the interpretation of the characteristics of each class, we named them ‘PCP-led Participatory Decision-making Model’ (the PCP-led model), ‘CM-led Membership Responsibility Model’ (the CM-led model), and ‘Stand-alone and Letter Referrals Model’ (the stand-alone model). Table 2 presents the probabilities of the variables in each model, while Table 3 provides a comparative description of them in plain language.

**Table 2:** Conditional probabilities of each variable for three-class latent class analysis.


CONCEPTS	VARIABLES	ITEMS	PCP-led (46.6%)	CM-led (32.8%)	Stand-alone (20.6%)

Team member	Number of professions	1	0.11	0.07	0.64

		2–3	0.28	0.40	0.36

		4≤	0.61	0.52	0.00

Member’s decision-making responsibility	Doctors	Yes	1.00	0.51	1.00

		No	0.00	0.49	0.00

	Care managers	Yes	0.25	0.97	0.09

		No	0.75	0.03	0.91

Organisation’s role	Hosting care conferences	Clinics	0.41	0.09	0.07

		Other organisations	0.46	0.73	0.18

		Not held	0.13	0.18	0.75

	Responsibility in care decisions	Clinics	0.78	0.22	0.67

		MCD	0.08	0.05	0.24

		CCSC	0.15	0.54	0.06

		LTC offices	0.00	0.19	0.03

Clinic’s inter-organisation communication	with MCD	Face to face	0.15	0.12	0.03

		In writing	0.56	0.68	0.58

		None	0.29	0.19	0.39

	with CCSC	Face to face	0.90	0.79	0.27

		In writing	0.08	0.13	0.20

		None	0.02	0.08	0.53

	with LTC offices	Face to face	0.76	0.83	0.14

		In writing	0.21	0.15	0.51

		None	0.03	0.02	0.35


MCD, Medical Centre for Dementia; CCSC, Community-based Comprehensive Support Centre; LTC, long-term care.

**Table 3:** Comparative description of the three models.


MODEL NAME	MODEL DESCRIPTION

PCP-led Participatory Decision-making Model	PCPs take the lead in making care decisions for individuals with early-stage dementia, with CMs partially contributing. Verbal communication serves as the primary mode of interorganisational collaboration. PCPs and/or CMs host interprofessional care conferences across organisations, during which team members share information to coordinate care and inform decision-making.

CM-led Membership Responsibility Model	CMs assume the primary responsibility for making care decisions for individuals with early-stage dementia. PCPs participate in the interprofessional care team and attend conferences to fulfil their responsibilities of providing information and make care decisions pertaining of the medical aspect. Clinics, CCSCs, and LTC offices collaborate closely through direct communication across organisations.

Stand-alone and Letter Referrals Model	PCPs are solely responsible for care decision for individuals with early-stage dementia, in which CMs are not involved. The practice does not leverage Interprofessional team or care conferences. Clinics’ interorganisational communication relies on exchanges of written referrals and replies.


The PCP-led model was characterised by the clinics that formed interprofessional teams consisting of more than four professionals (61%), with the PCPs largely responsible for the care decisions (100%) and some degree of shared responsibility with the CMs (25%). At the organisational level, the PCP-led model clinics communicated face-to-face with the CCSCs (90%) and the LTC offices (76%). These clinics led to organisational collaboration as they often hosted care conferences (41%) and were responsible for the care decision-making for each patient (78%). This model accounted for 46.4% of the entire sample.

The CM-led model clinics, accounting for 32.8%, formed interprofessional teams in which the CMs took significant responsibility for the care decisions (97%), whereas half of the PCPs did not have this responsibility. The inter-organisational communication patterns for this model were similar to those for the PCP-led model; however, the roles of the clinics differed. The CM-led model clinics rarely hosted care conferences (9%) and left the decision-making roles to the CCSCs (54%) and the LTC offices (19%) where the CMs were generally employed.

The stand-alone model clinics, constituting 20.6% of the clinics, exhibited highly distinct characteristics regarding team configuration and inter-organisational collaboration. Unlike the PCP-led and CM-led collaborative models, they did not have interprofessional teams (64%) or communication with the CCSCs (53%); their communication with the LTC offices was primarily written. Thus, the CMs’ contribution to the care decisions was minimal (9%).

Table 4 presents a comparison of the clinical structures in each model. The clinics with the PCP- and CM-led models tended to have more PCPs and nurses as well as longer perceived time for consultation with the nurses than the stand-alone model clinics. The PCP-led model clinics had significantly more patients with early-stage dementia than the stand-alone ones. The PCPs in PCP-led model clinics perceived that they spent significantly longer time consulting with patients with early-stage dementia than did those in the CM-led model clinics.

**Table 4:** Human resources and services of clinics in each model.


		PCP-led (n = 84)		CM-led (n = 55)		Stand-alone (n = 36)		p	1 vs 2	1 vs 3	2 vs 3

Human resources	Dr (full time), mean, SD	1.7	(1.2)	1.5	(1.2)	1.4	(1.1)	0.455			

	Dr (part time), mean, SD	2.8	(4.1)	1.6	(2.7)	1.4	(2.3)	0.052			

	NS (full time), mean, SD	2.9	(3.8)	2.2	(4.4)	1.1	(1.9)	0.056			

	NS (part time), mean, SD	1.2	(1.7)	1.7	(2.4)	1.1	(1.6)	0.191			

	SW, n,%	14	(16.7%)	4	(7.3%)	2	(5.6%)	0.109			

	CM, n,%	11	(13.1%)	6	(10.9%)	2	(5.6%)	0.477			

	PT/OT/ST, n,%	17	(20.2%)	10	(18.2%)	2	(5.6%)	0.13			

	Psych, n,%	8	(9.5%)	3	(5.5%)	2	(5.6%)	0.597			

Number of patients with early-stage dementia	<50	56	(67.5%)	38	(70.4%)	33	(91.7%)	0.027		*	

	50–99	12	(14.5%)	8	(14.8%)	0	(0.0%)				

	100<	15	(18.0%)	8	(14.9%)	3	(8.4%)				

Perceived time for Dr consultation	<10 min	24	(29.3%)	22	(40.7%)	16	(45.7%)	0.02	*		

	10–19 min	38	(46.3%)	27	(50.0%)	15	(42.9%)				

	20–29 min	15	(18.3%)	4	(7.4%)	3	(8.6%)				

	30 < min	5	(6.1%)	1	(1.9%)	1	(2.9%)				

Perceived time for NS consultation	0 min	45	(57.0%)	30	(57.7%)	26	(76.5%)	0.065			

	<10 min	14	(17.7%)	14	(26.9%)	6	(17.6%)				

	10–19 min	12	(15.2%)	5	(9.6%)	2	(5.9%)				

	20 < min	8	(10.1%)	3	(5.8%)	0	(0.0%)				

SW or CM consultation	Yes	11	(13.2%)	10	(18.2%)	1	(2.9%)	0.34			


PCP, primary care physician; CM, care manager; Dr, doctor; NS, nurse; SW, social worker; PT, physical therapist; OT, occupational therapist; ST, speech therapist; Psych, psychologist. P-values for overall and pair-wise comparisons were calculated using chi-square and Z tests for categorical variables; one-way ANOVA and two-sample t-test for continuous variables; and Kruskal-Wallis and Mann-Whitney U tests for ordinal variables. For the pair-wise comparisons, p-values were corrected with Bonferroni method. The symbol * indicates statistical significance for pair-wise comparisons.

Table 5 shows the results of the seven domains of support provision for people with early-stage dementia. Across all clinical models, cognitive function, physical health, and care planning were the most commonly provided services, with over 85% of the clinics offering support in these domains. By contrast, informational support, psychological health, and social health were the least commonly provided services, with 56.0–66.3% of the clinics reporting offering daily support in these domains.

**Table 5:** Daily practices of support provision in each model.


	TOTAL (n = 175)		PCP-led (n = 84)		CM-led (n = 55)		Stand-alone (n = 36)		p	1 vs 2	1 vs 3	2 vs 3

Cognitive function												

Assessment of cognitive function	166	(94.9%)	84	(100.0%)	52	(94.5%)	30	(85.7%)	0.003		*	

Medication for BPSD	164	(93.7%)	81	(96.4%)	53	(96.4%)	30	(85.7%)	0.052			

Physical health												

Assessment of physical health	165	(94.3%)	81	(96.4%)	53	(96.4%)	31	(88.6%)	0.174			

Fall risk intervention	151	(86.3%)	77	(91.7%)	47	(85.5%)	27	(75.0%)	0.051			

Care planning												

Medical care plan	160	(91.4%)	84	(100.0%)	49	(90.7%)	27	(79.4%)	<0.001		*	

Long-term care plan	153	(87.4%)	82	(97.6%)	46	(83.6%)	25	(71.4%)	<0.001	*	*	

Carer support												

Assessment of carers’ health status	144	(82.3%)	79	(94.0%)	46	(85.2%)	19	(54.3%)	<0.001		*	*

Carer counselling	100	(57.1%)	53	(63.9%)	32	(59.3%)	15	(42.9%)	0.105			

Informational support												

Patient basic information	116	(66.3%)	68	(81.0%)	35	(63.6%)	13	(36.1%)	<0.001		*	

Carer basic information	116	(66.3%)	71	(85.5%)	33	(61.1%)	12	(34.3%)	<0.001	*	*	

Psychological health												

Assessment of psychological health	119	(68.0%)	57	(67.9%)	38	(69.1%)	24	(66.7%)	0.97			

Post-diagnostic counselling	102	(58.3%)	53	(63.1%)	33	(60.0%)	16	(44.4%)	0.157			

Social health												

Assessment of social health	112	(64.0%)	63	(75.0%)	35	(64.8%)	14	(40.0%)	0.001		*	

Dementia café	98	(56.0%)	59	(70.2%)	29	(53.7%)	10	(28.6%)	<0.001		*	


PCP, primary care physician; CM, care manager; BPSD, behavioural and psychological symptoms of dementia. P-values for overall and pair-wise comparisons were calculated using chi-square and Z tests. For the pair-wise comparisons, p-values were corrected with Bonferroni method. The symbol * indicates statistical significance for pair-wise comparisons.

The PCP-led model showed a significantly higher likelihood of providing support than the stand-alone one in several areas: assessment of cognitive function (100% vs 85.7%), medical care planning (100% vs 79.4%), LTC planning (97.6% vs 71.4%), assessment of the carer’s health status (94.0% vs 54.3%), patient information support (81.0% vs 36.1%), carer information support (85.5% vs 34.3%), assessment of social health (75.0% vs 40.0%), and dementia café (70.2% vs 28.6%). Meanwhile, the CM-led model generally fell in the middle of the other two models regarding the proportion of support provision; however, it was significantly less likely to provide LTC planning and carer informational support than the PCP-led model; moreover, it was significantly more likely to assess the carers’ health status than the stand-alone one. The three models did not differ significantly in terms of support for psychological health.

## Discussion

Our findings indicate that case management based on the PCP-CM collaboration can be beneficial for people with early-stage dementia, as both PC-led and CM-led models tend to be more responsive to the characteristic needs at this stage, such as informational and social health support, than the stand-alone one.

### Different leadership in the PCP-CM collaboration for early-stage dementia

The two collaborative models were classified based on the combination of resources available in Japan’s Community-based Integrated Care System regulated by the governmental healthcare policy, where the PCPs work in their clinics and the CMs in the LTC offices and CCSCs [27]. They highlighted the differences in the leading roles in the PCP-CM collaboration. As both models were based on interdisciplinary collaboration in the community and exhibited the potential to offer comprehensive assessments, care planning, and care coordination for people with early-stage dementia and their families, we interpreted them as applications of case management.

Recent review articles on dementia care for primary care clinics have elaborated on the practice model typologies, including PCP-Case Management Partnership Models, that emphasise a case management approach orbiting around the PCP-CM collaboration [5,12,13]. However, how these collaborative models can be applied effectively is controversial [13,36], particularly in the context of delivering care to people with early-stage dementia [11,24]. For example, some have argued that solid leadership from the PCPs is crucial for case management [18], whereas others contend that responsible commitment from the CMs is necessary [36]. In the context of the Community-based Integrated Care System, where the primary framework for early-stage dementia care lacks defined collaboration methods or insurance reimbursement, the two different leadership styles largely reflect the stances of PCPs in early-stage dementia care. Our findings indicate that both leadership styles of PCP-CM collaboration, accounting for about 80% of clinics, can effectively accommodate the diverse care needs of individuals with early-stage dementia. Thus, it is reasonable for the system to maintain flexible collaboration methods within the context of preventive social care. However, the remaining 20% of certified dementia support PCPs did not leverage collaboration, despite its availability. This suggests the need for related strategies, such as providing financial incentives for PCP-CM collaboration, to encourage their participation in early-stage dementia care in the community.

### Scope of support provision for meeting the needs at an early stage

In this study, all clinical models reported relatively low rates of service delivery in the domains of informational support, psychological health, and social health, which are highly relevant to individuals with early-stage dementia, especially in the period following diagnosis [8]. These domains may have been less prioritised by the PCPs in this research, who primarily specialised in internal medicine, compared to the domains closely related to their speciality, such as physical and cognitive health. However, as more clinics in the two collaborative models, especially the PCP-led model, offered informational and social health support than those in the stand-alone one, a collaborative approach has the potential to improve practices to meet the characteristic needs at an early stage.

One exception was the psychological health domain, which showed consistently low provision rates across all the models. This could be attributed to the lower priority given to this domain. This finding highlights the need for incorporating more content on psychological care in the training course for Certified Dementia Support Doctors to assess and handle the psychological state of individuals with early-stage dementia. However, it is also plausible that limited community resources and ambiguity regarding where and by whom these services are offered may be contributing factors [34,37]. To improve early-stage dementia care in the community, it is essential for the primary care clinics to improve the practices of integrating and tailoring support and for the community to develop and clarify the necessary resources. By working together, these efforts can create synergy to improve care.

### Necessity of a task-sharing approach within clinics

The PCP-led model was found to be advantageous in delivering a wider range of care, especially compared to the stand-alone one. This may be due to the PCPs’ greater time and effort contributed to consultations, as they spent significantly longer time consulting the patients with dementia. Additionally, although the two collaborative models tended to include more nurses than the stand-alone one, the proportion of nurses engaged in consultation sessions did not differ significantly across the models. The PCPs argue that they are already stretched thin from responding to diverse patient populations [38]. Irrespective of the leadership of the collaborative care models, a task-sharing approach with other professionals in the community is required for contemporary post-diagnostic support for people with dementia [2,38]. Task sharing within clinics can be enhanced by involving nurses in consultation sessions for these people and their families. Nurses could suitably partly or broadly fulfil the role of case managers when a dedicated CM is unavailable [36].

## Limitations

This study has several limitations. First, the low response rate might have resulted in a less representative sample. For instance, the proportions of internists (70.6%) and psychiatrists (11.2%) in this study were different from the previous statistics of 48.9% and 27.6%, respectively [39]. However, latent class analysis provided a reasonable solution regarding the number of classes and reflected the reality of the existing practices. Additionally, considering that the sample size was close to the minimum required for rigorously conducting latent class analysis [40], we ran multiple analyses using different combinations of indicator variables to assess the robustness of the identified classes. Second, the data may have been biased because they were derived solely from the PCPs. The CM-led model was inferior to the PCP-led one in providing the LTC plans and carer information support, which are typically CMs’ responsibilities. Further research with unbiased data is needed to make direct comparisons between the two collaborative models with different leadership styles.

## Conclusion

In the framework of Community-based Integrated Care Systems where Certified Dementia Support Doctors engage, two distinct leadership styles emerge in PCP-CM collaborations for delivery care to individuals with early-stage dementia. While the PCP-led model may offer a broader range of care, it may require more time from PCPs during consultations. In the CM-led model, the PCPs’ contribution to the comprehensiveness of the services may be lower. Despite these differences, case management based on the PCP-CM collaboration proves beneficial for people with early-stage dementia compared to the Stand-alone model.

The study’s findings underscore two important lessons. Firstly, collaborative models may not be advantageous in addressing the psychological health needs of individuals with early-stage dementia. Second, some certified PCPs are hesitant to adopt PCP-CM collaboration in environments where CMs operate in external organisations. These lessons warrant further scrutiny to understand the underlying reasons for this hesitation and devise appropriate strategies to address it.

## Additional File

The additional file for this article can be found as follows:

10.5334/ijic.7726.s1 Appendix. Full list of daily practices in each model.
